# Muscle Lipid Metabolism: Role of Lipid Droplets and Perilipins

**DOI:** 10.1155/2017/1789395

**Published:** 2017-06-06

**Authors:** Pablo Esteban Morales, Jose Luis Bucarey, Alejandra Espinosa

**Affiliations:** ^1^Departamento de Tecnología Médica, Facultad de Medicina, Universidad de Chile, Santiago, Chile; ^2^CIDIS-AC, Escuela de Medicina, Universidad de Valparaiso, Valparaiso, Chile; ^3^Center for Molecular Studies of the Cell, Facultad de Medicina, Universidad de Chile, Santiago, Chile

## Abstract

Skeletal muscle is one of the main regulators of carbohydrate and lipid metabolism in our organism, and therefore, it is highly susceptible to changes in glucose and fatty acid (FA) availability. Skeletal muscle is an extremely complex tissue: its metabolic capacity depends on the type of fibers it is made up of and the level of stimulation it undergoes, such as acute or chronic contraction. Obesity is often associated with increased FA levels, which leads to the accumulation of toxic lipid intermediates, oxidative stress, and autophagy in skeletal fibers. This lipotoxicity is one of the most common causes of insulin resistance (IR). In this scenario, the “isolation” of certain lipids in specific cell compartments, through the action of the specific lipid droplet, perilipin (PLIN) family of proteins, is conceived as a lifeguard compensatory strategy. In this review, we summarize the cellular mechanism underlying lipid mobilization and metabolism inside skeletal muscle, focusing on the function of lipid droplets, the PLIN family of proteins, and how these entities are modified in exercise, obesity, and IR conditions.

## 1. Introduction

Obesity and type 2 diabetes mellitus (T2DM) have become hallmark pandemic events of our century, currently affecting 600 million [1] and 380 million adult people [2], respectively. These pathologies have a longstanding stablished relation, as obese patients are often diabetic as well [3, 4]. Increased free fatty acid (FFA) plasma levels are present in both pathologies [5, 6], and therefore, they have been conceived as a major link between obesity and T2DM.

T2DM is a disorder of variable etiology characterized by sustained hyperglycemia, with alterations of carbohydrate, fat, and protein metabolism [7]. In the case of T2DM, this overt hyperglycemia results from the reduced action of insulin on its target tissues, such as skeletal muscle, liver, and adipose tissue, at least on initial stages [4, 8, 9]. In this regard, the proper function of skeletal muscle is of paramount importance, given that it is involved in the clearance of 25% of plasmatic glucose in a basal, fasting state [8] and up to nearly 70–85% of plasmatic glucose in postprandial state [10, 11]. This substantial increment in skeletal muscle glucose uptake is the result of increased presence of the facilitative glucose transporter 4 (GLUT4) in the sarcolemma and T-tubule in response to insulin action. This transporter is basally located in intracellular vesicles and moves to and fuses with the plasma membrane as a result of insulin-mediated signaling [12, 13]. The particularities of glucose transport, kinetics, and mechanisms are beyond the scope of this review and can be read elsewhere [14, 15].

As mentioned before, alteration of FA metabolism is also an important feature of T2DM patients, as their plasmatic levels are often increased [5, 16]. However, different lines of evidence have indicated that accumulation of different lipidic entities inside muscle cells leads to insulin resistance. Increased ceramide [17], intramyocellular lipids (IMCLs) [18], diacylglycerol (DAG) [19, 20], and long-chain fatty acyl-CoA [21] levels have been negatively correlated with insulin action, depicting the importance of understanding the link between obesity and the lack of insulin response in skeletal muscle. The mechanisms involved in intracellular lipid accumulation and how these phenomena are involved in IR is relevant to understand the extent of obesity-induced damage in skeletal muscle. In this review, we begin with a comprehensive view of lipid metabolism in healthy skeletal muscle, covering uptake, metabolization, and storage. We then focus on the function of lipid droplets (LDs), an organelle responsible for both intracellular storage and trafficking of FAs between different cellular compartments, and provide information on how LDs contribute to insulin resistance in the obese state, with special interest on specific LD proteins, the PLIN protein family.

## 2. Overview of Lipid Metabolism in Healthy Skeletal Muscle

Skeletal muscle is responsible for the body's energy expenditure, participating in thermogenic functions, glucose and lipid uptake, and other metabolic processes. The fuel supply is obtained from metabolic machinery involving enzymatic pathways in charge of obtaining energy from glucose and FAs, through glycolysis and *β*-oxidation, respectively. These processes are dependent on substrate availability [22, 23].

**Figure 1:**
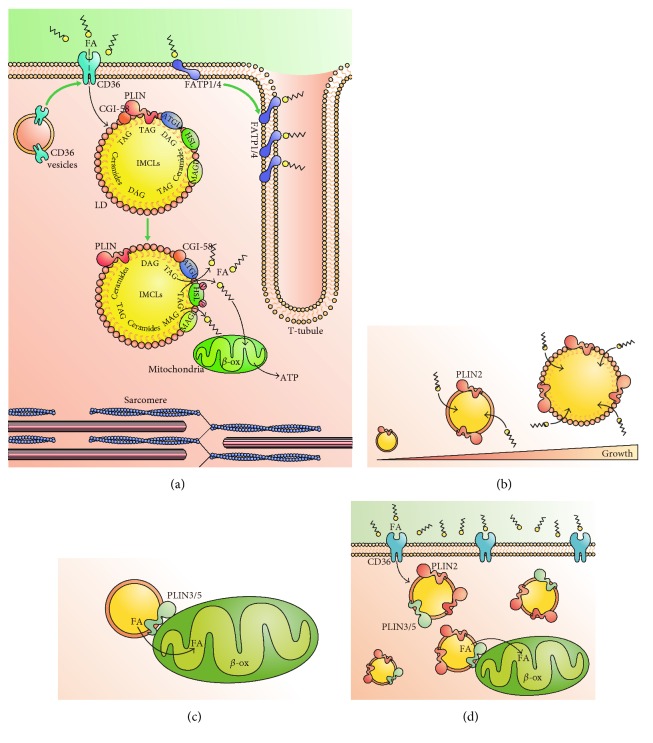
FA flux in skeletal muscle and role of perilipins. (a) Schematic representation of FA uptake and deposition in lipid droplets (LD). FA uptake is mediated by FAT/CD36, located in the plasmalemma. FATP1/4 are located in the plasmatic membrane and cooperate with FA intake and metabolism. Movement of CD36 to the plasmatic membrane and an increment in FATP1/4 can be triggered by insulin or contraction (green arrow, see text for details). Once inside the cell, FAs can be accumulated in LDs as acyl-glycerides (TAG, DAG). Perilipins (PLINs) coat the LD membrane, along with the lipases ATGL and HSL and the coactivator CGI-48. In response to contraction (green arrow), ATGL activity increases, as a result of dissociation from PLINs, while HSL is activated by PKA-dependent phosphorylation. This leads to increased FA flux to cytosol and mitochondria, undergoing further *β*-oxidation (*β*-ox) and ATP synthesis. (b) Main function of PLIN2 in skeletal muscle. PLIN2 coats LD and promotes FA intake, leading to increased size of LD. (c) PLIN3 is part of LD mitochondria contact sites, promoting efficient transfer of FA from LD to mitochondria for oxidation. (d) In cases of excess plasmatic FAs, such as obesity and T2DM, FAs uptake into skeletal muscle is increased. Higher levels of PLIN2 promote an increase in both size and number of LDs. Furthermore, increased levels of PLIN5 foster FA metabolization inside mitochondria.

Lipolysis is the process in which triacylglycerides (TAGs) are broken down to produce FFAs. Increased FA turnover is triggered by various stimuli, including *β*-adrenergic agonists and exercise [24, 25]. FA uptake in the muscle is dependent on metabolic demands and lipid availability. Once inside the cell, FAs enter the oxidative process, TAG synthesis, or if uptake exceeds metabolization, they undergo accumulation in confined compartments, often LD. Acute lipid oversupply produces inhibition of glucose oxidation, and mitochondria preferentially switch from carbohydrate to FA utilization, depicting the high degree of metabolic flexibility in skeletal muscle [26, 27]. In fact, the sole alteration of FA entrance machinery levels is able to modulate FA oxidation rate [28] indicating a high level of metabolic interregulation.  Figure 1 depicts the key points regarding FA flux inside skeletal muscle cells, as will be discussed in the following sections.

### 2.1. Lipid Uptake

FAs move from plasma into skeletal fibers using different proteins, such as FA binding proteins [29] and FA transport proteins (FATPs) [30]. All of these proteins are upregulated by classic stimuli that are often associated to skeletal muscle: insulin and contraction [31–33]. For instance, FA translocase (also known as cluster of differentiation 36, CD36), which translocates from cytoplasm to plasma membrane in specialized vesicles, is able to relocate in response to muscle contractions [34]. Furthermore, CD36 mRNA and protein levels are upregulated by high-lipid diet feeding, increasing FA uptake in skeletal muscle [35]. As for FATPs, these proteins are expressed in a tissue-specific manner, with FATP1, FATP4, and FATP6 variants being the predominant forms in skeletal muscle [36]. Its function is still debated, as they have been also shown to possess enzymatic activity (as acyl-CoA synthetases), besides its transport function [37]. FATP1 is present in T-tubules, and its overexpression has been shown to increase FA oxidation in skeletal muscle [38, 39]. After contraction, FATP4 and CD36 are both increased in the sarcolemma, while insulin stimulation induces the translocation of FATP1 and FATP4 to the T-tubules, increasing FA uptake [38].

Once FAs enter the skeletal fiber, they have different fates depending on the metabolic status of the cells. In resting condition, plasma FAs are driven into TAG synthesis as the first destination instead of being moved to the mitochondria for oxidation [40].

### 2.2. Lipid Storage

Increased lipid deposition in skeletal muscle develops when skeletal fiber FA uptake outpaces FA oxidation. An excessive lipid flux into the skeletal muscle is a factor that influences the accumulation of lipid intermediates, which in turn produces lipotoxic stress [41, 42]. Lipid excess generates fatty infiltrations, also called intermuscular adipose tissue, and IMCLs. Evidence suggests that intermuscular adipose tissue is related to the aging process, loss of muscle strength, and decrease in muscle insulin sensitivity [43]. IMCLs are stored in LDs localized between the sarcomeres and adjacent to mitochondria [44], providing an energy pool used in acute and chronic exercise, as will be discussed in the next sections.

IMCLs are composed of triacylglycerol, diacylglycerol (DAG), long-chain acyl-CoA, and ceramides and both DAGs and ceramides are implicated in muscle lipotoxic effect [45]. As mentioned earlier, IMCL accumulation is associated with alterations in insulin signaling [18]. It is worth noting, however, that IMCLs are not always related to obesity. In healthy subjects, women can accumulate 57% more lipids than men in skeletal tissue, without being obese [46]. Hoeg et al. suggested that there is not a clear association between TAG content and impairment of insulin-stimulated muscle glucose uptake [46]. Indeed, accumulation of LDs is often present in endurance athletes [47], who have functional insulin signaling. This phenomenon is known as the “athlete's paradox,” and its mechanism remains unclear [48].

Several lines of evidence show that elevated lipid intermediates such as DAGs and ceramides are associated with impaired insulin signaling in skeletal muscle [19, 20, 49–51]. Curiously, DAGs are also higher in trained athletes, which were associated with improved insulin sensitivity [52], suggesting that DAG content is not always indicative of insulin signaling derangement.

### 2.3. Lipolysis in Skeletal Muscle

There are three lipases expressed in skeletal muscle that are responsible for the breakdown of TAGs: monoacylglycerol lipase, adipose triglyceride lipase (ATGL), and hormone-sensitive lipase (HSL) [53]. ATGL catalyzes the first step of TAG lipolysis in skeletal muscle from humans and mice, resulting in the release of one fatty acid molecule. Monoacylglycerol lipase is responsible for the hydrolysis of monoacylglycerol, releasing glycerol and FAs. Overexpression of ATGL in skeletal muscle cells generates an increase in lipolysis and in the expression of the transcription factor PPAR*δ*, suggesting a role of ATGL in mitochondrial biogenesis [54]. This in turn is associated with increased oxidative capacity in skeletal muscle [54, 55]. Endurance training leads to increased ATGL levels, enhancing intramuscular lipolysis, mainly in type I oxidative fibers [56]. On the contrary, a decrease in ATGL expression characterizes aged muscle, which is accompanied by defects in the antioxidant response and sarcopenia [57].

ATGL is activated by comparative gene identification-58 (CGI-58), a protein member of *α*/*β*-hydrolase fold enzyme family [58]. These proteins are located on the surface of LDs and mitochondria [59], CGI-58 being preferentially expressed in oxidative muscle, as cardiac and soleus muscle [60]. The increase in lipolytic activity results from direct interaction between CGI-58 and ATGL [58]. CGI-58 knockdown reduces lipolysis and incorporation of FAs into TAGs, along with reducing mitochondrial membrane potential [60]. Likewise, muscle-specific inactivation of CGI-58 in mice induces skeletal steatosis but only in oxidative muscle [61]. It is not surprising then that CGI-58 mutations produce lipid storage diseases, such as the Chanarin-Dorfman syndrome, which is characterized by neutral lipid accumulation in skeletal muscle and ichthyosis [62]. Interestingly, Xie et al. found that diminished CGI-58 levels improve glucose tolerance and insulin sensitivity in mice fed with high-fat diet (HFD). The authors explain these findings by suggesting that storing fat in glycolytic muscle is detrimental, whereas it is healthy in oxidative muscle [61].

Similar to ATGL, HSL catalyzes TAG hydrolysis to release FAs into the cytoplasm. Like CGI-58, HSL is highly expressed in type I oxidative fibers of skeletal muscle, and it is activated by adrenergic stimulation and contraction [63, 64]. Both stimuli are capable of regulating HSL activity through PKA- and AMPK-dependent phosphorylation, thus modulating the breakdown of TAGs from IMTG [65].

## 3. Role of Lipid Droplets and Perilipins

As shown in the previous sections, FA metabolism in skeletal muscle requires a tight balance between the uptake and usage processes, to avoid accumulation of detrimental lipid intermediaries. In this regard, LDs play a pivotal role in maintaining intracellular lipid homeostasis.

LDs are intracellular vesicle-like organelles composed mainly of neutral lipids, including TAGs and sterol esters (Figure 1(a)), and are present in different cellular types, with adipose tissue and skeletal muscle being the most studied [66]. Its formation is a consequence of different metabolic processes, such as lipid storage, lipid exchange between organelles, and cell signaling. LDs are limited by a phospholipid monolayer, which includes phosphatidylcholine, phosphatidylethanolamine, and phosphatidylinositol [67]. This composition is similar to the endoplasmic reticulum (ER) membrane, supporting the idea that LDs are a specialized domain of the ER [68].

LDs are covered with different members of lipid droplet-associating protein family, referred as the PAT family [69]. This family is composed of perilipins (PLINs), S3-12, and TIP47, among other proteins, which vary on tissue-specific expression and their constitutive or differentiated permanence on LD surface [70, 71]. The most characterized members of the PAT family are PLINs, which are discussed in detail below.

Perilipin family is composed by 5 members (PLIN1–5) that play a major role in the control of TAG hydrolysis and lipolysis in adipose tissue, with a less clear role in skeletal muscle. PLIN1 is expressed in adipocytes and steroidogenic cells but not in skeletal muscle [72, 73], and therefore, its function is not further discussed.

On the other hand, PLIN2 is highly expressed in adipose tissue and skeletal muscle in both rodents and humans [74]. The levels of this protein correlate positively with LD content in skeletal muscle [74], where it can interact with ATGL [75] and HSL [76]. PLIN2 is necessary for both differentiation and regulation of lipolysis in adipose tissue, but its role in skeletal muscle remains unclear. Overexpression of PLIN2 results in increased intracellular TAG storage and larger and more numerous LDs in tibialis anterior muscle [77]. In agreement, downregulation of PLIN2 in cultured myotubes prevented oleate-induced lipid droplet storage, suggesting its involvement in LD stabilization [77]. Evidence suggests that PLIN2 participates in IMCL synthesis and LD growth (Figure 1(b)), but it seems that it is not implicated in skeletal muscle lipolysis, as PLIN2 is not phosphorylated under adrenergic or contractile stimulation [76]. Interestingly, PLIN2-coated LDs increase with age and are associated with lack of muscle strength [78]. This is interesting, as aging is associated with insulin resistance [79].

Unlike PLIN2, the role of PLIN3 in skeletal muscle is not clear. Pharmacological and genetic activation of AMP-activated protein kinase leads to increased gene expression of PLIN3 in skeletal muscle, resulting in higher IMCL content [80]. Conversely, PLIN3 levels in muscle biopsies from healthy patients are positively correlated with whole-body oxidative capacity, while PLIN3 knockdown results in decreased FA oxidation [81]. Subcellular localization of PLIN3 may play a role in its function, as it has been observed that endurance training, but not electrically induced contraction, induces PLIN3 expression and its association with mitochondria in rats (Figure 1(c)) [82]. Whether species-specific function or intracellular localization is responsible for this discrepancy is still unknown.

Little is known about PLIN4 in skeletal muscle. This perilipin is expressed in skeletal muscle, heart, and adipose tissue, and it is preferentially located in LDs containing cholesterol esters [83]. Of all perilipins, *Plin4* mRNA is the most expressed in vastus lateralis biopsies from healthy individuals, and its levels are higher in slow- than fast-twitch muscle [84]. Unlike PLIN3, PLIN4 expression is reduced in response to prolonged endurance training [84].

PLIN5 is found both on the surface of LDs and in the cytoplasm, and it is transcriptionally regulated by PPAR*δ* in skeletal muscle. PLIN5 is highly expressed in oxidative tissue such as cardiac and skeletal muscle [85]. Laurens et al. conclude that PLIN5 has an important role in lipolysis, facilitating FA oxidation in response to contraction and increased metabolic demand [86]. PLIN5 is involved in the communication between the LDs and the mitochondria, presumably to facilitate the direct transfer of FFAs released during lipolysis (Figure 1(c)). In fact, there is close structural proximity between PLIN5-decorated LDs and mitochondria [87] and PLIN5 overexpression leads to increased transcription of mitochondrial biogenesis, electron transport chain complexes, and FA oxidation genes [88]. PLIN5 overexpression increases both expression and serum concentration of fibroblast growth factor 21, a major insulin- and exercise-responsive myokine [89]. Furthermore, its overexpression can induce gene expression of factors involved in the ER stress response in order to preserve mitochondrial function [89].

## 4. Lipid Droplet Remodeling in Skeletal Muscle after Contraction

Skeletal muscle is a plastic tissue susceptible to different stimuli but mainly adrenergic stimulation and contraction. Studies have been designed to evaluate PLIN distribution after contraction-induced muscle lipolysis in skeletal muscle, in order to understand the physiological role of PLINs in metabolism. The mechanisms that regulate exercise-induced lipolysis in skeletal muscle are poorly understood, and reports indicate that they may be more complex than lipolysis in adipose tissue. Electrical stimulation is used to induce acute contractile stimulation in isolated fibers [90]. Interestingly, LD and PAT associations are differentially modified in adipose tissue and skeletal muscle when chronic stimulation is applied, such as endurance training [91].

LDs, PLIN2, and PLIN5 are located in the subsarcolemmal region during rest. PLIN2 and PLIN5 are found in LDs and in the cytosol. Neither PLIN2 nor PLIN5 localization changes after contraction in soleus muscle, even when LD content is decreased after electrical stimulation [92]. As mentioned above, ATGL and CGI-58 are necessary for the activation of HSL in the regulation of lipolysis. In this context, both PLIN2 and HSL translocate to LDs after electrically stimulated acute contraction [93]. In fact, exercise rapidly triggers protein kinase A-dependent HSL activation in humans, promoting FA release [65, 94]. ATGL-CGI-58 protein interaction increases and ATGL-PLIN2 decreases after electrical stimulation [75]. The same acute stimulation is capable of increasing mitochondrial PLIN5 content in rats [76]. However, chronic exercise does not change PLIN5 level in muscle biopsies obtained from patients subjected to training intervention [84]. PLIN4 mRNA, in turn, is decreased after exercise programs consisting of combined strength and endurance training for 12 weeks [84].

## 5. Findings on Insulin Resistance in Skeletal Muscle

### 5.1. Obesity as a Main Cause of Insulin Resistance

Despite the great knowledge that has been generated in the recent years by studying the molecular mechanisms involved in the generation of IR as a result of obesity, several questions remain unresolved. Obesity is characterized by enhanced accumulation of FAs in adipose tissue, liver, and skeletal muscle, and HFD feeding is an accepted model for obesity and IR. Long-term HFD feeding produces an increase in LD content and PLIN5 expression in skeletal muscle [95]. As discussed above, one of the mechanisms involved in HFD-dependent IR is the presence of toxic lipid intermediates as a result of lipid management derangements.

### 5.2. Oxidative Stress

An intracellular pro-oxidative environment has been reported in skeletal muscle from obese and insulin-resistant individuals. We have shown that insulin-resistant mice show increased insulin-stimulated H_2_O_2_ release, and a decreased reduced-to-oxidized glutathione ratio [96]. Some reports suggest that PLIN5 plays a protective role against oxidative burden in the heart, suppressing excess ROS production by sequestering FAs in TAGs [97], but there is no direct evidence in skeletal muscle showing that ROS are involved in the gene expression of PLINs.

### 5.3. Lipotoxicity

Lipotoxicity leads to the damage of organelles that are necessary for intracellular metabolic control, due to an excessive accumulation of lipid intermediates such as lipid-derived DAGs and ceramides [41]. As described before, it has been proposed that the generation and accumulation of these lipid intermediates alters insulin-stimulated glucose uptake [19, 49–51]. FA intermediates activate serine/threonine kinases that impair the ability of the insulin receptor to activate downstream targets, as IRS-1. This leads to decreased translocation of GLUT4 and therefore reduced glucose uptake into skeletal muscle cells [98–100]. Skeletal muscle uses LDs as a protective mechanism against the accumulation of these lipid intermediates. As for the role of PLIN2 in lipotoxicity, it has been reported that PLIN2 knockdown prevents intramyocellular TAG storage, while PLIN2 overexpression augments myocellular fat storage and neutral TAG accumulation in LDs [77]. DAG levels are not increased in this model. Accordingly, PLIN2 overexpression reverts palmitate-induced impairments in insulin signaling [77]. Increased PLIN2 expression inhibits GLUT-mediated glucose uptake into skeletal cells apparently via the retention of SNARE fusion machinery proteins for vesicular fusion at the plasma membrane. This suggests that PLIN2 may play an important role in regulating skeletal insulin response [101]. In fact, PLIN2 is increased in skeletal muscle from rats with genetic-induced diabetes, which might be considered as a compensatory mechanism to deal with excessive lipid load (Figure 1(d)) [74]. Interestingly, skeletal muscle from patients with T2DM had lower PLIN2 gene expression compared to the skeletal muscle of obese control subjects, although a trend to increased protein levels was observed [102].

Studies from two separate groups report that overexpression of PLIN5 in skeletal muscle results in increased LD size and richness in TAGs but does not impair insulin sensitivity [88, 89]. In fact, T2DM patients' muscle biopsies do not show significant differences in PLIN5 levels compared to matched control patients [74]. These findings may be explained by the reports that PLIN5 overexpression in skeletal muscle is capable of protecting the cells against lipotoxicity by increasing the amount of esterified lipid chains into LDs [103]. DAG accumulation is reduced in PLIN5 overexpressing myotubes treated with palmitate, which elevates ceramides and DAG content [103]. On the other hand, fasting is a physiological IR model, in which PLIN5 has been proposed to decrease lipotoxicity by promoting interaction of LDs with mitochondria. Fasting produces an increase in insulin resistance and mitochondrial dysfunction associated with higher presence of PLIN5 in LDs (Figure 1(d)). The authors hypothesized that this effect could be explained by an expanded capacity for inert lipid storage [104].

### 5.4. Autophagy

Autophagy is a cellular process that generates the degradation of damaged cytoplasmic organelles and proteins [105]. LDs contribute to the initiation of autophagy, promoting autophagosome biogenesis through phosphatidylcholine generation from TAG hydrolysis [106].

Mitochondrial function has been shown to be impaired in IR-related diseases. Maintaining autophagy flux is necessary to prevent accumulation of dysfunctional mitochondria and conserve the skeletal muscle mass [105]. Recently, it has been proposed that LDs are linked to the dynamic mitochondrial process. In skeletal muscle, the presence of damaged mitochondria generates oxidative stress and apoptosis, both of which can produce atrophy and muscular weakness [107]. Skeletal muscle from IR individuals has a higher degree of oxidative stress and toxic lipid intermediates accumulation, both conditions associated to mitochondrial damage [108]. Dynamin-related protein-1 (Drp-1) participates in mitochondrial fragmentation, which may result in mitochondrial dysfunction and IR [109]. Specifically, it was described that CGI-58 promotes mitochondrial fission through upregulation of Drp-1 expression producing mitochondrial fragmentation. Reports also showed that CGI-58 overexpression leads to significantly higher basal levels of both autophagy and mitophagy in the C2C12 myotube cell line [110]. Intracellular lipidic stores can be broken down through the process of autophagy, a catabolic pathway that ultimately delivers specific cargo to lysosomal degradation. It has been suggested that PAT- and LD-interacting proteins are part of the autophagy machinery, driving the process of LD recycling [111, 112]. Elegant genetic and imaging approaches have shown that LDs are engulfed by autophagosomes and then associated with lysosomes for degradation [113]. Furthermore, this process is accompanied by degradation of PLIN2 and PLIN3 in the LDs, allowing for effective recruitment of the autophagic machinery and metabolism of the TAG contained within [114]. The LDs' role in autophagy in skeletal muscle is an open field of research.

### 5.5. Insulin Resistance Independent of Impaired Insulin Signaling

A great proportion of specialized literature suggest that IR in skeletal muscle is a result of impaired insulin signaling. Considering that GLUT4 appears to be responsible for most of both contraction- and insulin-stimulated glucose transport, any defect produced in GLUT4 subcellular trafficking could be responsible of glucose uptake impairment, which is the ultimate step in insulin action.

Upon contraction or insulin stimulation, GLUT4-containing vesicles translocate, dock, and fuse with the plasma membrane through the action of N-ethylmaleimide-sensitive factor attachment protein receptors, known as SNARE fusion machinery proteins [115, 116]. In this regard, PLIN2 overexpression inhibits GLUT4-mediated glucose uptake, with the apparent mechanism being retaining SNARE proteins for vesicular fusion at the plasma membrane, suggesting that PLIN2 may play an important role in regulating glucose uptake [101]. Furthermore, SNARE proteins have been found in LDs and mitochondria in skeletal muscle [117], which suggests that other PLIN isoforms, as PLIN3 or PLIN5, might also be involved. GLUT4-vesicule fusion to plasma membrane is also dependent on Ca^2+^ signaling [118, 119], and therefore, disturbances in Ca^2+^ homeostasis could be a factor involved in insulin resistance, independent of impaired insulin signaling.

## 6. Conclusions

Until now, questions regarding the specific functions of different PLINs in skeletal muscle remain unanswered. The fact that two completely different physiological conditions, as IR and exercise, results in the accumulation of LDs in skeletal muscle continues to be a topic of debate. Could it be that different patterns of PATs in LDs causes this difference? Another possibility is that the intracellular distribution of the LDs differs in these two conditions, as their interactions with organelles such as mitochondria and lysosomes is becoming an apparent regulatory mechanism in LD dynamics. Despite the current uncertainties, it may be conceived that PLIN protein function in skeletal muscle is similar to that observed in adipose tissue: regulators and promoters of FA intake into LDs, and physical mediators that facilitate its further metabolization in mitochondria.

Throughout this review, we have depict the different and multiple processes involved in lipid metabolism in skeletal muscle. The myriad of stimuli that act on skeletal muscle (such as insulin, contraction, and adrenergic stimulation) and the tone of activation (whether it is acute or chronic); all of them can impinge on lipid metabolic pathways. In consequence, a detailed study on all of these variables is necessary in order to more precisely conclude the role of LDs in skeletal muscle in both healthy and disease states.
